# Melatonin Alleviates the Suppressive Effect of Hypoxanthine on Oocyte Nuclear Maturation and Restores Meiosis via the Melatonin Receptor 1 (MT1)-Mediated Pathway

**DOI:** 10.3389/fcell.2021.648148

**Published:** 2021-04-15

**Authors:** Jing Wang, Zhiyong Zhuo, Xiao Ma, Yunjie Liu, Jing Xu, Changjiu He, Yao Fu, Feng Wang, Pengyun Ji, Lu Zhang, Guoshi Liu

**Affiliations:** ^1^Beijing Key Laboratory of Animal Genetic Improvement, Key Laboratory of Animal Genetics and Breeding of the Ministry of Agriculture, National Engineering Laboratory for Animal Breeding, College of Animal Science and Technology, China Agricultural University, Beijing, China; ^2^Beijing Keao Xieli Feed Co., Ltd., Beijing, China

**Keywords:** melatonin, MT1, oocyte maturation, meiosis, cAMP

## Abstract

It is well known that hypoxanthine (HX) inhibits nuclear maturation of oocytes by elevating the intracellular cAMP level, while melatonin (MT) is a molecule that reduces cAMP production, which may physiologically antagonize this inhibition and restore the meiosis process. We conducted *in vitro* and *in vivo* studies to examine this hypothesis. The results showed that 10^–3^ M MT potentiated the inhibitory effect of HX on mouse oocyte meiosis by lowering the rate of germinal vesicle breakdown (GVBD) and the first polar body (PB1). However, 10^–5^ M and 10^–7^ M MT significantly alleviated the nuclear suppression induced by HX and restored meiosis in 3- and 6-week-old mouse oocytes, respectively. We identified that the rate-limiting melatonin synthetic enzyme AANAT and melatonin membrane receptor MT1 were both expressed in oocytes and cumulus cells at the GV and MII stages. Luzindole, a non-selective melatonin membrane receptor antagonist, blocked the activity of MT on oocyte meiotic recovery (*P* < 0.05). This observation indicated that the activity of melatonin was mediated by the MT1 receptor. To understand the molecular mechanism further, MT1 knockout (KO) mice were constructed. In this MT1 KO animal model, the PB1 rate was significantly reduced with the excessive expression of cAPM synthases (*Adcy2*, *Adcy6*, *Adcy7*, and *Adcy9*) in the ovaries of these animals. The mRNA levels of *Nppc* and *Npr2* were upregulated while the genes related to progesterone synthesis (*Cyp11a11*), cholesterol biosynthesis (*Insig1*), and feedback (*Lhcgr*, *Prlr*, and *Atg7*) were downregulated in the granulosa cells of MT1 KO mice (*P* < 0.05). The altered gene expression may be attributed to the suppression of oocyte maturation. In summary, melatonin protects against nuclear inhibition caused by HX and restores oocyte meiosis via MT1 by reducing the intracellular concentration of cAMP.

## Introduction

Adenylated cyclases (ADCYs) catalyze the conversion of ATP to cyclic adenosine 3′,5′-monophosphate (cAMP) (Hanoune and Defer, 2001), an important second messenger that participates in multiple cellular functions (Gold et al., 2013; Zhang et al., 2020). Hypoxanthine (HX) (Downs et al., 1985; Eppig et al., 1985; Eppig and Downs, 1987; Ma et al., 2003), NPPC/NPR2 (Zhang et al., 2010; Kiyosu et al., 2012), and some other small molecular peptides in follicular fluid secreted by granulosa cells (Huang et al., 2013; He et al., 2016; Zhang et al., 2017) can inhibit phosphodiesterase (PDE) activity and maintain a high intracellular concentration. This high level of cAMP inhibits oocyte meiosis (Pirino et al., 2009) and allows the oocytes to be in a meiotic arrest state and keeps them in the GV stage in the follicles.

Phosphodiesterases play an important role in maintaining the balance of cAMP concentrations (Brunton, 2003; Beltejar et al., 2017; Rohrig et al., 2017). Among PDEs, PDE3A mainly degrades cAMP within the oocyte. In the luteinizing hormone (LH) secretion peak, PDE3A is activated to metabolize cAMP and to recover meiosis (Richard et al., 2001). At a relatively low level of LH (for example, before the LH secretion peak), cGMP can enter oocytes through gap junctions and inhibit PDE3A activity. In the presence of PDE3A, a lower cGMP concentration in oocytes leads to GVBD (Norris et al., 2009; Vaccari et al., 2009; Zhang et al., 2010).

Regarding the impact of cAMP on oocyte maturation, melatonin is a well-known molecule that regulates intracellular cAMP levels in a variety of cell types. The effect of melatonin (MT) on oocyte maturation has been extensively studied. For instance, MT can promote the maturation of fish eggs by inducing the hormone molt-inhibiting hormone (MIH). In mammals, MT significantly promotes oocyte maturation and improves embryo quality in cattle, pigs, and sheep (Zhao et al., 2016; Tian et al., 2017; Wang et al., 2017). The signaling pathway of MT mediated by its membrane receptor is the inhibition of cAMP production. This effect was first reported in frog melanocytes (White et al., 1987) and subsequently in many mammalian tissues, including the pituitary and cerebral arteries (Roka et al., 1999).

In addition, MT effectively reduces endoplasmic reticulum (ER) stress (Park et al., 2018), protects porcine oocytes *in vitro* maturation from heat stress (Li et al., 2015), and improves the quality of frozen thawed oocytes in mouse ovaries (Hemadi et al., 2009). MT1 and MT2 melatonin membrane receptors have been detected in bovine oocytes, and their activation by MT significantly improves the oocyte cleavage rate and blastocyst rate during *in vitro* maturation (Sampaio et al., 2012). Based on this evidence, we speculate that MT may cooperate with LH to relieve nuclear suppression and restore meiosis of oocytes. These activities of MT may be mediated by receptors to regulate the intracellular concentration of cAMP. To confirm these hypotheses, we examined whether MT application can prevent HX inhibition of oocyte meiosis and investigated the underlying mechanisms since HX is an established PDE inhibitor that elevates intracellular cAMP.

## Materials and Methods

### Animal Treatment

Wild-type Kunming White and C57BL/6J mice (*Mus musculus*) were purchased from the Laboratory Animal Center of Academy of Military Medical Science (Beijing, China). *Mtnr1a* knockout (MT1 KO) C57BL/6J mice were reconstructed via the CRISPR/Cas9 system (Zhang et al., 2019). Mice were kept in the facility at China Agricultural University according to the guidelines for laboratory animals. All animal treatment procedures were approved by the Animal Care Committee of China Agricultural University (CAU), and the protocol number for the mouse study was AW21119102-2-1. Mice were housed under controlled temperature (22–26°C) and lighting (16 hours light/8 hours darkness) with food and water *ad libitum*.

### Chemicals

TRIzol reagent was purchased from Life Technologies Corporation. Oligo dT and reverse transcriptase enzyme reverse transcription reagents were purchased from Fermenters. PCR primers were subscribed by Invitrogen. PCR Mix and ddH_2_O were purchased from Tiangen Company. MEMα was purchased from Gibco (United States). Pregnant mare serum gonadotropin (PMSG) and human chorionic gonadotropin (hCG) hormone were obtained from Ningbo Second Hormone Factory (Ningbo Hormone Products Co., Ltd., China). Fetuin (Merck, Germany), MT, and other test reagents were purchased from Sigma (St. Louis, MO, United States) unless otherwise stated.

### Mouse Oocyte Collection and Culture

For the *in vivo* study, mice aged 3 weeks (before sexual maturity) and 6 weeks (after sexual maturity) were used. Mice were sacrificed by cervical dislocation 44 h after they were primed with 10UI PMSG, and the oocytes were immediately collected from the ovaries. Briefly, the follicles were punctured under a stereomicroscope using a 29G needle fixed to a 1 mL disposable syringe and aspirated into M2 medium supplemented with 4 mM HX (Wang et al., 2004). Cumulus oocyte complexes (COCs) were washed three times using M2 and cultured in 50 μL drops of MEMα medium under mineral oil. Mouse oocytes were matured in an atmosphere of 5% CO_2_ at 37°C. After 4 and 18 h of incubation, germinal vesicle breakdown (GVBD) and first polar body (PB1) ovulation were evaluated. COCs were cultured under the following conditions: HX-free medium, 4 mM HX medium, and HX medium with MT (10^–3^, 10^–5^, 10^–7^ and 10^–9^ M). To identify whether the melatonin membrane receptor is involved in the signal transduction pathway, 10^–7^ M luzindole (a melatonin membrane receptor antagonist) (Wang et al., 2014) was added to the culture medium of some groups. In addition, in some animals, 10 UI hCG was injected 44 h after PMSG injection. Then, after 16 h of *in vivo* maturation, the oocytes were removed from the oviduct, and the number of superovulations and PB1 in oocytes of WT and MT1 KO mice were recorded.

### RNA Isolation and qPCR

Total RNA was isolated from granulosa cells, cumulus cells, and ovaries using TRIzol reagent. First-strand cDNA was produced by using oligo dT primers and reversed transcriptase according to the manufacturer’s instructions. RNA was quantified using a NanoDrop 2000 spectrophotometer (Thermo Scientific) by measuring the ratio of the absorbance at 260 nm to the absorbance at 280 nm. The real-time qPCR reactions consisted of SYBR Green (10 μL), forward and reverse primers (0.5 μL and 0.5 μM), template cDNA (2 μL, 500—1,000 ng) and ddH_2_O added up to a total volume of 20 μL. The qPCR was performed using the primers as in Supplementary Table S1. Annealing, an extension, and total number of cycles for the three transcripts were 60, 53, and 58°C for 30 s, respectively, and 72°C for 50 s, 35 cycles. Normalization was performed using actin and beta (*Actb*) as a control. Gene sequences were found on the NCBI website^1^, and Primer Premier (version 5.00) was used for primer design. Primer sequences are listed in Supplementary Table S1. The experiments were replicated at least three times.

### Detection of cAMP and cGMP

Both cAMP and cGMP were detected via an ELISA kit. Eighteen hours after *in vitro* maturation, cumulus cells in each group were digested with 0.1% hyaluronidase, and then oocytes in the cumulus cells were washed with M2 operating liquid containing 0.2 mM isobutylmethylxanthin (IBMX). Each group of oocytes was incubated with 0.1 M hydrochloric acid on ice for processing for at least 10 min. After a short freezing, the samples were preserved at −80°C in liquid nitrogen. For analysis, the samples were thawed on ice and centrifuged at 600 g. For 10 min, the supernatant was collected and dried at 60°C. The samples were reconstituted with 200 μL Assay Buffer 2. The samples were detected according to kit instructions.

### Immunofluorescence

After the GV and MII COCs were isolated, they were placed in paraformaldehyde for 40 min, 0.1% Triton X-100 in PBS for 20 min, and 0.3% BSA in PBS for 1 h and then incubated with anti-AANAT antibody (1:100 dilution; ab3505; Abcam), anti-MT1 antibody (1:200 dilution; ab203038; Abcam), and anti-MT2 antibody (1:200 dilution; ab203346; Abcam) overnight at 4°C. COCs were further incubated with fluorescent secondary antibody (1:1,000 dilution; 111-165-144; Jackson) at room temperature for 2 h, washed with PBS, and then incubated with nuclear staining (bisbenzimide H33342 trihydrochloride, Hoechst 33342; 1:1,000 dilution; 14533; Sigma) for 5 min. A laser confocal microscope platform (Leica TCS SP8, Leica Microsystems, Germany) was used to take immunofluorescent photos in which excitation light with wavelengths of 488 and 594 nm was used.

### Statistical Analysis

All experiments were performed at least three times. Data were expressed as the mean ± S.E.M. The values were analyzed by ANOVA followed by Tukey’s *post hoc* comparisons. Statistical analyses were performed using the computer software SPSS 19.0. *P* < 0.05 was considered significant.

## Results

### The Effects of Melatonin on GVBD and PB1 in Mouse Oocytes in the Presence of HX

**FIGURE 1:**
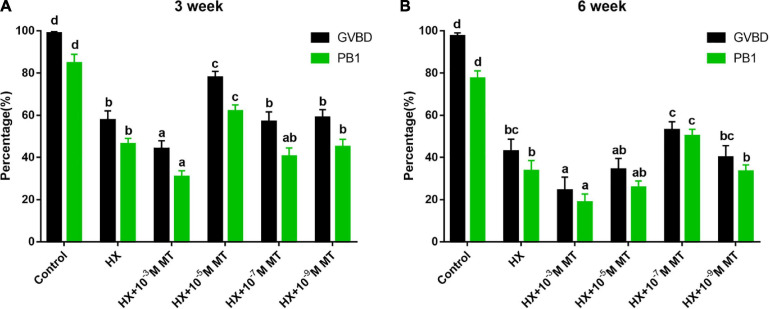
The effect of melatonin on the meiosis of mouse oocytes in the presence of HX. GVBD and PB1 during oocyte maturation. **(A)** Three-week-old. Control (*n* = 153), HX (*n* = 153), HX + 10^–3^ M, MT (*n* = 159), HX + 10^–5^ M, MT (*n* = 154), HX + 10^–7^ M MT (*n* = 158), HX + 10^–9^ M MT (*n* = 161). **(B)** Six-week-old mice. Control (*n* = 152), HX (*n* = 148), HX + 10^–3^ M MT (*n* = 149), HX + 10^–5^ M MT (*n* = 158), HX + 10^–7^ M MT (*n* = 152), HX + 10^–9^ M MT (*n* = 153). HX, 4 mM hypoxanthine; MT, melatonin. Data represents the mean with SEM. Different superscript letters (a–d) in each column present significant differences. (*P* < 0.05) determined by one-way ANOVA followed by Tukey *post hoc* comparisons.

Under HX treatment, oocyte meiosis was suppressed. This effect was indicated by the significantly lower GVBD and PB1 rates than those in the control groups without HX (*P* < 0.05; Figure 1). MT treatment at a concentration of 10^–^3 M significantly lowered the GVBD and PB1 rates compared with those in the other groups (*P* < 0.05; Figure 1). MT at 10^–5^ M significantly increased the GVBD and PB1 rates of oocytes collected from 3-week-old mice compared to control groups (77.8 ± 2.9% vs 57.7 ± 4.4%, *P* < 0.01; 61.9 ± 3.0% vs 46.3 ± 2.7%, *P* < 0.01; Figure 1A). For oocytes collected from 6-week-old mice, similar phenomena were observed in the MT-treated groups, and the maximum effects were found in the MT 10^–7^ M group (Figure 1B).

### The Expression of MT Receptors and AANAT During Oocyte Maturation

**FIGURE 2:**
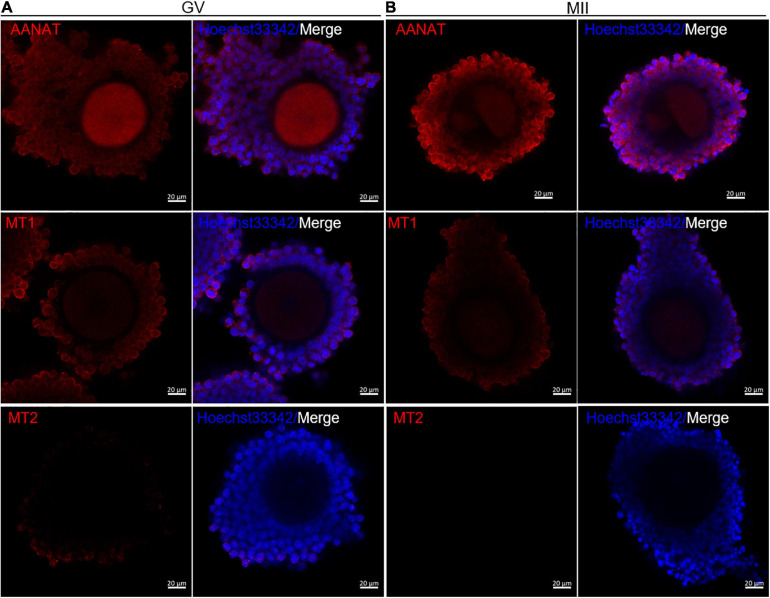
Expression of the melatonergic system during meiotic maturation in mouse oocytes. Immunofluorescence of AANAT, MT1 and MT2 in cumulus cells and oocytes at the GV stage **(A)** and MII stage **(B)**. Bar = 20 μm.

The immunofluorescence results showed that the melatonin synthetic enzyme *AANAT* and melatonin membrane receptor *MT1* were expressed during mouse oocyte maturation. *AANAT* and *MT1* were all expressed in cumulus cells and oocytes in both GV and MII stages (Figure 2). However, *MT2* was only slightly expressed in the cumulus of GV COCs (Figure 2). The above results suggest that the effects of melatonin on oocyte maturation may be mediated by MT1.

### Effects of MT1 on Nuclear Suppression Caused by HX

**FIGURE 3:**
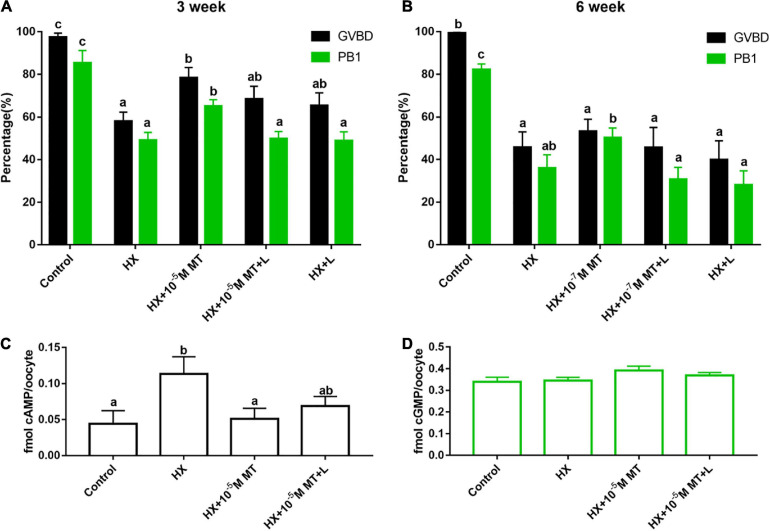
Effects of melatonin receptors on the recovery of meiosis in mouse oocytes and the levels of cAMP and cGMP. **(A)** Three-week-old; control (*n* = 119), HX (*n* = 117), HX + 10^–5^ M MT (*n* = 122), HX + 10^–5^ M MT + L (*n* = 117), HX + L (*n* = 120). **(B)** Six-week-old mice: control (*n* = 100), HX (*n* = 100), HX + 10^–7^ M MT (*n* = 104), HX + 10^–7^ M MT + L (*n* = 99), and HX + L (*n* = 104). **(C,D)** Effects of luzindole on cAMP and cGMP levels in oocytes. Levels of cAMP and cGMP in oocytes from different groups were tested by ELISA. **(C)** cAMP: Control (*n* = 419), HX (*n* = 416), HX + 10^–5^ M MT (*n* = 425), HX + 10^–5^ M MT + L (*n* = 419). **(D)** cGMP: Control (*n* = 320), HX (*n* = 320), HX + 10^–5^ M MT (*n* = 320), HX + 10^–5^ M MT + L (*n* = 310). HX, 4 mM hypoxanthine; MT, melatonin; L, 10^–7^ M luzindole. Data represent the mean with SEM. Different superscript letters (a–d) in each column represent significant differences vs respective groups. (*P* < 0.05) determined by one-way ANOVA followed by Tukey *post hoc* comparisons.

For 3-week-old mouse oocytes, the beneficial effect of melatonin on nuclear suppression induced by HX was blocked by luzindole (L). MT and its receptor inhibitor luzindole supplemented the GVBD rate in the HX + 10^–5^ M MT + L group and was lower than that in the HX + 10^–5^ M MT group, but the difference did not reach significance (68.3 ± 6.2% vs 78.4 ± 4.8%, *P* > 0.05). However, the PB1 rate in the HX + 10^–5^ M MT + L group was significantly lower than that in the HX + 10^–5^ M MT group (65.0 ± 3.0% vs 49.7 ± 3.4%, *P* < 0.05). As the control, the GVBD rate in the HX + L group was not different than that in the HX group (65.3 ± 6.0% vs 57.9 ± 4.4%, *P* > 0.05) (Figure 3A).

Similar effects were observed in 6-week-old mouse oocytes as in 3-week-old mouse oocytes under all the treatments (Figure 3B).

### Effects of Melatonin on cAMP and cGMP Levels Under the Influence of HX

Hypoxanthine significantly increased the cAMP level in oocytes compared to the control (0.113 ± 0.0240 vs 0.044 ± 0.0183 fmol, *P* < 0.05). Melatonin treatment significantly reduced the cAMP level compared to that of the HX group (0.050 ± 0.0148 vs 0.113 ± 0.0240 fmol, *P* < 0.05; Figure 3C). Luzindole partially blocked the effects of melatonin on cAMP production but reached significance (0.068 ± 0.0133 vs 0.050 ± 0.0148 fmol, *P* > 0.05; Figure 3C). The cGMP levels were not significantly changed among the groups (*P* > 0.05; Figure 3D).

### Effects of MT1 KO on the Maturation of Mouse Oocytes *in vivo*

**FIGURE 4:**
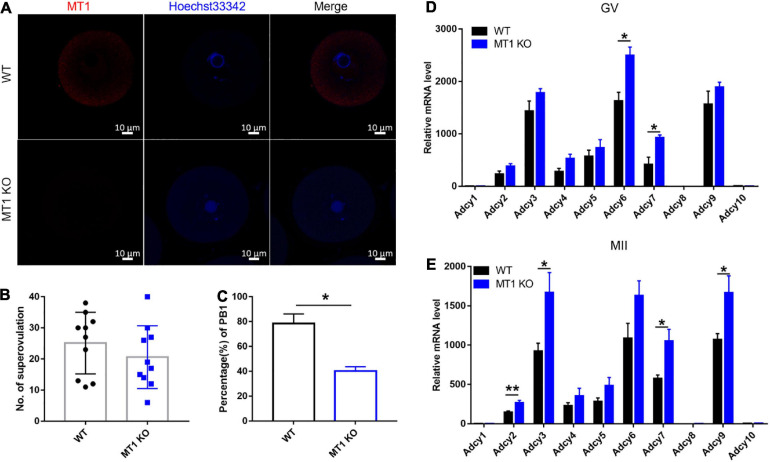
Effects of MT1 KO on mouse oocyte maturation and cAMP synthesis ability of the ovary. **(A)** Images of immunofluorescence of MT1 at the GV stage in WT and MT1 KO oocytes. Bar = 10 μm. **(B,C)** The number of superovulated oocytes and the rate of PB1 in WT and MT1 KO oocytes. No. of superovulation: *n* = 7; PB1: *n* = 4. **(D,E)** The mRNA level of cAMP synthases in the ovaries of WT and MT1 KO mice detected by qPCR. Data represent the mean with SEM. (*P < 0.05, **P < 0.01) vs its respective group, determined by one-way ANOVA followed by Tukey *post hoc* comparisons (*n* = 5).

At 44 h after PMSG injection, GV oocytes were collected for analyses. MT1 expression was not detected in GV oocytes from MT1KO mice, as expected (Figure 4A). In the KO mice, the number of ovulations exhibited no significant difference compared to the WT mice (Figure 4B); however, the rate of PB1 of oocytes matured *in vivo* was significantly decreased in KO mice compared to the WT (*P* < 0.05; Figure 4C).

### Effects of MT1 KO on cAMP Synthase in Mouse Ovary

The results showed that the expression of cAMP synthesis enzymes functioning at the GV stage of the ovary, including adenylate cyclase 6 (*Adcy6*) and *Adcy7*, and functioning at the MII stage of the ovary, including *Adcy2*, *Adcy7*, and *Adcy9*, in MT1 KO mice was significantly upregulated compared to that in WT animals (*P* < 0.05; Figures 4D,E). The other adenylate cyclase enzymes that synthesized cAMP did not change significantly among the groups.

### Effects of MT1 KO on Granulosa Cell Function

**FIGURE 5:**
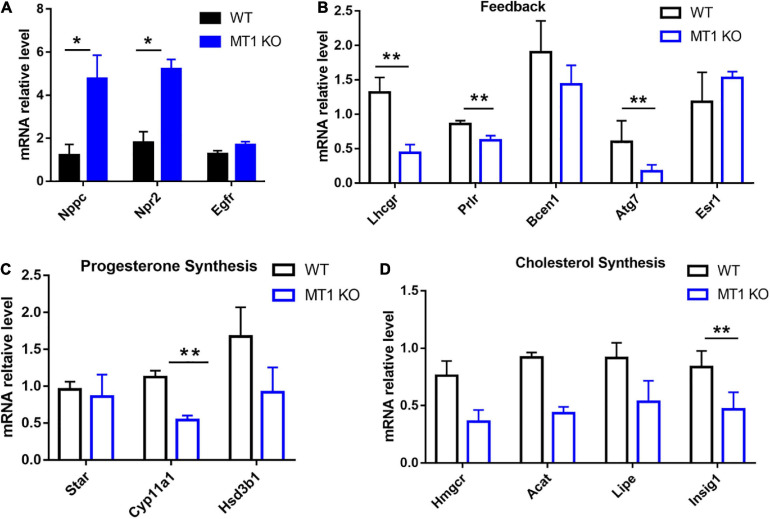
Effects of MT1 KO on the expression of *Nppc*/*Npr2* and genes related to progesterone synthesis. *Nppc*/*Npr2* are genes regulating oocyte maturation and progesterone synthesis in mouse GV granulosa cells. **(A)** *Nppc*/*Npr2* and *Egfr*. **(B)** Gene-related feedback. **(C)** Gene-related progesterone synthesis. **(D)** Gene-related cholesterol biosynthesis. Data represent the mean with SEM. (*P < 0.05, **P < 0.01) vs respective groups determined by one-way ANOVA followed by Tukey *post hoc* comparisons (*n* = 3).

The qPCR results showed that the expression of *Nppc/Npr2* was significantly upregulated in GV stage granulosa cells collected from MT1 KO mice compared to WT mice (*P* < 0.05; Figure 5A). The expression of *Egfr* was not significantly changed between MT1KO and WT mice (*P* > 0.05; Figure 5A). The results also showed that genes related to progesterone synthesis, *Cyp11a1* (cytochrome P450, family 11, subfamily a, and polypeptide 1) (*P* < 0.01; Figure 5C), cholesterol biosynthesis *Insig1* (insulin induced gene 1) (*P* < 0.01; Figure 5D) and feedback (*Lhcgr*, luteinizing hormone receptor; *Prlr*, prolactin receptor; *Atg7*, autophagy-related 7; *P* < 0.01; Figure 5B), were significantly downregulated in the granulosa of MT1KO mice compared to WT mice. The expression of other genes tested showed no significant alterations between the MT1KO and WT mice (Figure 5).

## Discussion

Oocyte maturation is regulated by a number of factors. Identifying the mechanisms of oocyte maturation is a critical step for improving the quality of oocyte *in vitro* maturation. Under *in vivo* conditions, many factors can lead to oocyte meiotic arrest in follicles. Among these factors, HX (Eppig et al., 1985; Ma et al., 2003) and certain peptides (Zhang et al., 2010) have the capacity to maintain oocytes at the stage of meiotic arrest. Thus, HX is often used to suppress oocytes during *in vitro* maturation.

Melatonin is also an important factor that regulates female reproductive activities. These include regulating sex hormone expression and ovarian function and promoting oocyte maturation and early embryonic development (Reiter et al., 2009). However, the mechanisms of MT in the mammalian oocyte meiotic resumption process have not been fully understood to date. MT has been demonstrated to be present in the follicular fluid of humans, pigs, and other animals (Brzezinski et al., 1987; Shi et al., 2009; Tamura et al., 2009). This finding suggests that MT may play an important role in the oocyte maturation process.

Wang et al. (2004) reported that high concentrations of melatonin of 7.1 × 10^–7^–3.5 × 10^–4^ M inhibit FSH-induced GVBD, while moderate physiological doses of melatonin promote it. For example, melatonin at a concentration of 10^–6^ M boosted mouse oocyte maturation (Salimi et al., 2014). The results from the current study were consistent with these reports. We observed that MT at 10^–5^ and 10^–7^ M significantly promoted oocyte maturation and meiosis of the GV, which were suppressed by HX. It was also found that age also played a role in the sensitivity to the different concentrations of melatonin. For example, to erase the inhibitory effect of HX on the COCs of superovulation, 3-week-old mice require a higher concentration of melatonin than 6-week-old mice (10^–5^ M vs 10^–7^ M). This requirement may be related to the different qualities of superovulated oocytes in mice of different ages, and it may also be caused by the different levels of melatonin receptors on COCs of different ages. This effect will be studied in the future.

We also observed that MT at a high concentration of 10^–3^ M potentiated the inhibitory effects of HX on oocyte maturation. The suppressive effects of high levels of melatonin may be related to its effects on NO production. MT is a nitric oxide synthase (NOS) inhibitor that can inhibit the production of NO, and a suitable concentration of NO is required for activating COCs (Bu et al., 2003; Bu et al., 2004). A high concentration of melatonin oversuppresses NO formation and thus negatively impacts oocyte meiosis. However, we hypothesized that the beneficial effects of melatonin on oocyte maturation and mitosis would be attributed to its effects on the levels of intracellular cAMP or cGMP and other second messengers. This mechanism has not been investigated up to date. It is well known that the effect of melatonin on cAMP is mediated by its membrane receptors (Browning et al., 2000; Sethi et al., 2008; Renzi et al., 2013).

MT1 activation inhibits forskolin-induced cAMP production through suppression of adenylate cyclase activity (i.e., binding Gi protein) (Witt-Enderby and Dubocovich, 1996; Brydon et al., 1999), thereby inhibiting the activation of PKA and cAMP response element binding protein (cAMP responsive element-binding protein, CREB) phosphorylation (Witt-Enderby et al., 1998). The MT2 receptor is also capable of inhibiting cAMP and cGMP production (Sharkey and Olcese, 2007). In this study, MT1 expression but not MT2 expression was identified in granulosa cells, cumulus cells and oocytes in mice. In this consideration, MT2 may not play a major role in oocyte maturation, but MT1 does. The melatonin receptor antagonist luzindole blocks the effect of melatonin on oocyte maturation, which strongly suggests that MT1 is the mediator of MT activity. HX is an inhibitor of cAMP degradation that maintains high levels of cAMP; thus, it has suppressive effects on oocyte maturation. In contrast, melatonin reduces cAMP production by MT1 activation; thus, melatonin can physiologically antagonize the suppressive effects of HX on oocyte maturation.

To investigate the effects of MT1 further, we constructed MT1 KO mice. In this animal model, *in vivo* oocytes exhibited a significantly reduced PB1 rate compared to WT mice, indicating reduced oocyte maturation and further suggesting the importance of melatonin and MT1 in female reproductive physiology. MT1 KO mice are expected to have elevated cAMP synthesis in the ovaries, and this elevated cAMP level causes insufficient initiation of oocyte meiosis as seen in the HX animal model.

Previous studies have also confirmed that the melatonin receptor is involved in mitochondrial function (Ahluwalia et al., 2018; Qi and Wang, 2020). Gene expression analyses showed that MT 1KO significantly upregulated the expression of the oocyte maturation inhibitory gene *Nppc/Npr2*, which functions at the mitochondrial level. These results provide molecular mechanisms by which melatonin and its MT1 influence oocyte maturation and mitosis for the first time. Another important aspect observed in the study is the potential association of melatonin and MT1 with LH. LH plays a pivotal role in oocyte maturation (Fan et al., 2008; Zamah et al., 2010; Arroyo et al., 2020; Chen et al., 2020; Liu et al., 2020). LH is similar to melatonin, which can lower the concentration of cAMP within the oocyte but has different mechanisms than melatonin (Sethi et al., 2008; Muhlbauer et al., 2011; Lyga et al., 2016). This feature can lead the combination of melatonin and LH to produce synergistic effects on cAMP production and oocyte maturation. Our results suggest that melatonin can promote LH generation via MT1 activation. First, LH is generated in the mitochondria. MT1 KO may affect the production of LH by affecting mitochondrial function since melatonin and LH are both synthesized in mitochondria. Second, MT1 KO significantly downregulated the expression of genes related to progesterone synthesis (*Cyp11a*1), cholesterol biosynthesis (*Insig1*), and feedback (*Lhcgr*, *Prlr*, and *Atg7*) in oocytes, and all these genes were associated with LH synthesis and located in the mitochondria (Tan and Hardeland, 2020). In the current study, although we did not detect the LH level in the MT1 KO mice, the results supported this speculation. In conclusion, we identified that the melatonin synthetic enzyme *AANAT* and melatonin membrane receptor *MT1* are all expressed in GV and MII oocytes. These observations indicate that mouse oocytes have the capacity to synthesize melatonin, which is consistent with previous reports (He et al., 2015). The locally generated melatonin can act on MT1 in oocytes to function as an autocrine promoter of oocyte maturation. A major mechanism is that melatonergic system activation reduces the intracellular level of cAMP in oocytes to promote their maturation. Thus, melatonin and its receptor MT1 are critical factors for oocyte quality and maturation under *in vitro* and *in vivo* conditions.

## Data Availability Statement

The datasets presented in this study can be found in online repositories. The names of the repository/repositories and accession number(s) can be found in the article/Supplementary Material.

## Ethics Statement

The animal study was reviewed and approved by Animal Care Committee of China Agricultural University.

## Author Contributions

GL designed the work. JW, ZZ, XM, YL, JX, CH, YF, FW, and LZ performed the experiments and analyzed the data. JW and ZZ wrote the manuscript. PJ and GL revised the manuscript. All authors contributed to the article and approved the submitted version.

## Conflict of Interest

ZZ was employed by company Beijing Keao Xieli Feed Co., Ltd. The remaining authors declare that the research was conducted in the absence of any commercial or financial relationships that could be construed as a potential conflict of interest.

## Footnotes

1 https://www.ncbi.nlm.nih.gov/
